# Validation of the STERIS Amsco 630LS steam sterilizer autoclave for inactivation of category a medical waste from patients with high-consequence infectious diseases

**DOI:** 10.1017/ice.2023.188

**Published:** 2024-02

**Authors:** Jessica A. Tarabay, Jill S. Morgan, Colleen S. Kraft, Mary Elizabeth Sexton

**Affiliations:** 1 Emory Healthcare, Atlanta, Georgia; 2 Division of Infectious Diseases, Emory University School of Medicine, Atlanta, Georgia; 3 Department of Pathology and Laboratory Medicine Emory University School of Medicine, Atlanta, Georgia

## Abstract

Hospitals caring for patients with high-consequence pathogens may need to safely manage large volumes of category A waste. Using biological indicators to assess for successful sterilization, autoclave cycle parameters that would inactivate 4 categories of waste were identified and validated utilizing a STERIS Amsco 630LS Steam Sterilizer.

Historically, care for patients with high-consequence infectious diseases (HCIDs) has been limited to biocontainment units (BCUs) that managed the challenges of providing complex clinical care, including waste handling.^1^ However, recent cases of viral hemorrhagic fevers (VHF) and the global mpox outbreak (in which clade identification was delayed)^2^ underscore the importance of healthcare facilities beyond traditional BCUs being prepared to handle category A waste.

Guidelines surrounding category A waste handling require it to be inactivated on site via incineration or autoclave inactivation or transported to an off-site facility that can perform inactivation compliant with federal regulations.^3^ Sites for waste incineration are rare, and transport of untreated infectious waste is expensive and logistically challenging, given potential volumes of patient waste.^4,5^ For example, the most critical patient with Ebola virus disease at Emory University Hospital generated 1,312 bags of waste needing disposal (personal communication). As a result, many regional treatment facilities for HCIDs have chosen to inactivate waste on site using an autoclave.^6^

Guidance for pathogen inactivation suggests that a goal temperature of 121°C be maintained for 30 minutes,^4^ and Flinn et al^7^ described the need for site-specific validation for different waste types, including goal sterilization temperatures. Centers with BCUs have previously described methods for autoclaving category A waste and noted challenges with protocol validation, including (1) 1–2 biological indicators placed within sealed bags sometimes did not pass; (2) BIs placed within solidifier used to stabilize liquid waste did not pass; and (3) different cycle types, temperatures, and durations were necessary to achieve kill depending on the consistency and volume of waste.^7,8^ Due to these challenges, a prior validation study led to recommendations for using different cycle types for dry waste, damp linen, and liquid waste, avoiding the use of solidifiers, and opening bags prior to placing them in the autoclave.^9^ Although these modifications inactivated biological indicators, opening waste bags presents a potential for contamination to staff and the environment, and the solidifier is useful for safe movement of liquid waste. To maximize safety to personnel, we explored the validation of autoclave parameters that ensure inactivation of all waste types while maintaining bag closure.

## Methods

### Equipment

We utilized a Steris Amsco 630LS Steam Sterilizer (Steris, Mentor, OH), with Ellab ValSuite (Ellab-Hilleroed, Denmark) data loggers deployed alongside Steris submergible SA Spordex self-contained biological indicators (SCBIs) to record and validate the efficacy of the sterilization parameters for surrogate waste.^9^

### Waste composition and handling

We identified 4 types of waste based on prior studies^7^: general patient waste, linens, liquids, and laboratory or sharps waste. Surrogate waste was created to emulate the types and volumes anticipated in BCU patient care. We reviewed limited published data on waste packaging techniques,^7,10^ and we securely packaged the waste according to our BCU protocol to ensure adequate inactivation using standard safety measures. Simulated waste was placed into one biohazard bag (Fisherbrand Autoclave bag 01-814D, Thermo Fisher, Ottawa, Ontario). SCBIs and data loggers were placed within the waste in locations where sterilization might be challenging (eg, inside a coverall sleeve; center of a solidified suction canister). Bags were then closed via a gooseneck knot and tape (3M Comply Steam Indicator Tape, 3M Maplewood, MN) and placed inside a second biohazard bag, also goosenecked and taped closed.

### Waste inactivation trials

Initial autoclave cycle parameters of purge time, sterilize time, pulse vacuum, number of pulses, and dry phase were selected for each waste type.^6,8,10^ Each cycle contained 3–6 bags of a single waste type. If all biological indicators passed for a load, we assessed whether it was possible to increase waste volumes or bag numbers or to decrease cycle time to maximize efficiency. If any biological indicators failed, autoclave parameters were adjusted before repeating the cycle. Once parameters were identified and all biological indicators passed, the same process was repeated to obtain 3 successful cycles (Supplementary Material online).

## Results

**Figure 1.**
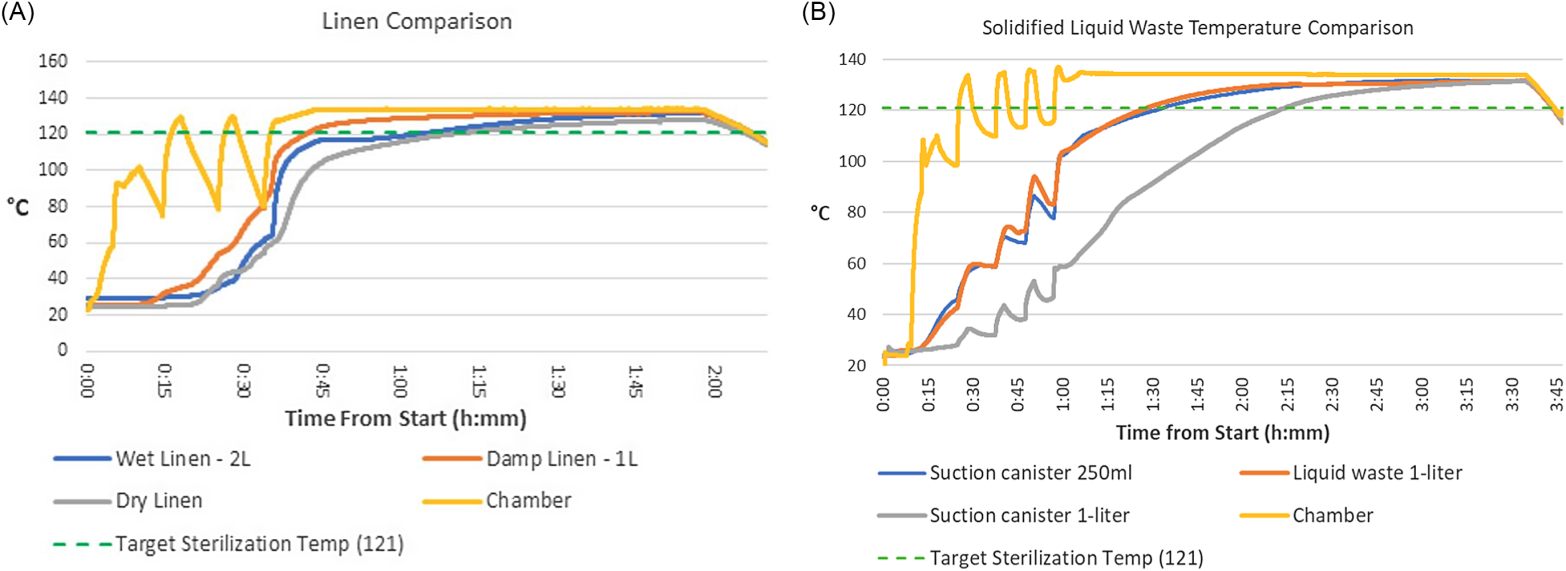
(A) Linen cycle temperature comparison. (B) Solidified liquid waste temperature comparison.

Successful cycle parameters were identified for each waste type, with kill of all BIs present within the load on 3 successful cycles (Table 1). For linen, the cycle time increased from baseline after the data loggers revealed disparities in target temperature acquisition depending on the presence and amount of moisture added to the linens (Fig. 1A). The liquid waste with solidifier demonstrated a delayed rise in temperature, particularly liquids within a container rather than a biohazard bag (Fig. 1B). Although the autoclave itself reached and maintained a sterilization temperature of 132°C, loads run using a data logger demonstrated that the liquids took longer to reach an acceptable minimal temperature (121°C) and were maintained at that temperature for a shorter true sterilization time. It took 9 attempts to identify final parameters for liquids, modifying the number of vacuum pulses, steam charge, and sterilization time. Laboratory or sharps waste similarly required 9 total loads to achieve three successful biological indicator inactivations, with the sterilization time doubled to 60 minutes.

**Table 1.** Optimized Cycle Parameters by Waste Type

Parameters	General Waste	Linens	Liquids	Sharps
Purge time, minutes	5	5	5	4
Pulse vacuum (in Hg)/pulse charge (PSIG)	15/26	15/26	20/26	20/26
No. of pulses	4	3	4	3
Sterilization temperature, °C	132	132	132	132
Sterilization time, minutes	45	75	150	60
Vacuum dry, in Hg	10	10	10	10
Dry time, minutes	15	15	5	5
Exhaust speed	Fast	Slow	Slow	Slow
Total cycle time	1 h 35 min	2 h 40 min	4 h 10 min	2 h 20 min

Note. Hg, millimeters mercury; PSIG, pounds per square gauge.

Visual inspection of the bags at the conclusion of the cycles confirmed that they all either ruptured or their tape closures had released during the cycle. The position of the bags on the rack, the position of the inner closure relative to the outer closure, and the tape used did not impede the opening of the bags during the cycle.

## Discussion

Our findings indicate that relying on standard autoclave settings for temperature and sterilization time will not reliably result in the inactivation of hazardous biological materials because the autoclave chamber temperature does not necessarily reflect the actual temperature of the contents being processed. Regardless of the autoclave type, institutions that want to deactivate category A waste on site need a robust quality assurance program, including the use of SCBIs, to ensure the safety and efficacy of their autoclave settings. Autoclave parameters need to be selected with particular attention to waste type, load size, and waste packaging with SCBIs and data loggers before treatment.^2^

This study had several limitations. The type of validation we performed has limitations, including the use of simulated waste and the potential for novel pathogens in the future with different cycle parameter requirements. We generated waste simulated to approximate the volume, density, and types of waste encountered during the care of critically ill patients with VHF. However, future patients may require care, equipment, and/or supplies that were not anticipated. Additionally, the biological indicators used followed the compliance standards for the sterilization of medical equipment, using goal parameters of 30 minutes at 121°C for each cycle. Future pathogens may require more extensive cycle times or temperatures, and it will be important for facilities to adjust accordingly as new pathogens emerge, including re-validation of autoclave settings.

Finally, institutions that choose to autoclave category A waste will need to assess the risks and benefits of separating waste types. As we identified in this study, separating waste allows some loads to be processed quickly, which helps with autoclave throughput if a patient generates high volumes of waste. However, it also requires multiple trash containers within a patient room, staff awareness of what belongs in each waste category and where it should go, and potentially temporary storage space for bags of each waste type until there is enough for a full autoclave load. Therefore, decisions about waste handling require interdisciplinary collaboration with infection prevention, facilities, and environmental services teams, who can review all protocols to evaluate safety.
